# Parental Attitudes and Perceptions Towards Vaccines

**DOI:** 10.7759/cureus.7657

**Published:** 2020-04-13

**Authors:** Zuhal Gundogdu

**Affiliations:** 1 Pediatrics, Kocaeli University, Kocaeli, TUR

**Keywords:** parental attitudes, perceptions, vaccines, vaccination policy

## Abstract

Objective

Success of any vaccination program and uptake of children's vaccines largely depends upon the parents' perceptions and attitudes. This study provides a snapshot of parents' views towards vaccines included in the nationally-funded vaccination program, together with two unfunded vaccines in Kocaeli, Turkey.

Methods

Data were gathered from a convenience sample through a questionnaire that explored the socio-demographic factors of the parents and the vaccination status of their children. The survey content was based on Health Belief Model. Mann-Whitney test was used for comparisons as there is no prior information regarding data distribution and the dependent variable is represented on an ordinal scale. Out of 300 parents who brought their children into the pediatrics polyclinics, 262 parents responded positively and took part. The questionnaires were filled out by mothers alone 67.6 % (n=177), 28.6 % (n=75) by fathers and mothers (both present), 3.1% (n=8) by fathers alone and the remaining 0.8% (n=2) were filled by other relatives.

Results

While the majority of diseases prevented by the vaccines were perceived to be serious, pneumococcal infection and chickenpox were considered to be less serious and there was no strong opinion for the rotavirus vaccine. The main reasons that influenced parents' decisions towards unfunded vaccines were advice from a pediatrician and the cost. Lastly, demographic factors such as family income, mothers' education and job types of mothers were important in contrast to previously published studies.

Conclusions

The acceptance of a new vaccine depends on a complex interaction of factors, but parents' perceptions may vary considerably. The main factors influencing parental acceptance are the availability of information and the cost. Therefore, for a new vaccination program to succeed, it should be funded by the government and a specific public health education program should be undertaken.

## Introduction

Vaccines and vaccination programs are among the greatest public health accomplishments of the 20th century as they have made a significant impact on the morbidity and mortality of children worldwide [1-3]. The World Health Organization (WHO) recommends routine childhood immunization to be considered in countries where a given disease is a relatively important public health and socioeconomic problem, where the vaccine is available and affordable, and where high and sustained vaccine coverage can be achieved [4].

In Turkey, the decision for a vaccine to be included in the national immunization program is taken by the Ministry of Health, in consultation with an advisory board [5]. The national immunization program coverage rate increased from 95% in 2007 to 96% in 2009 and 2010 [5-6]. The vaccination coverage rate for Kocaeli, where this study was undertaken, was in line with this rate according to the City Health Authority. The routine childhood primary immunization schedule in Turkey currently includes eleven recommended infectious agents: hepatitis B, diphtheria, pertussis, tetanus, polio, Haemophilus influenza type b (Hib), tuberculosis (BCG), pneumococcus, measles, mumps, and rubella. The seven-valent, conjugated pneumococcal vaccine was added into the nationally funded vaccination program only for children born after May 2008.

The Turkish national immunization program did not include varicella and rotavirus vaccines at the time of this study. These vaccines were only available in private clinics for a fee and they are generally recommended in private clinics. For those who decide to have their child immunized at their own expense, some get partial reimbursements through private health insurance companies. However, hepatitis A at 18 and 24 months and varicella vaccine at 12 months for children born after 2012 had been added into the nationally funded immunization program in 2013.

It has been reported that parental perception of the severity of the disease was one factor in determining the uptake rate of vaccination, together with information supplied by health professionals, in order to make an informed decision [7-9]. A study of parents’ perceptions and attitudes specifically concerning varicella vaccine found that the mothers’ job types, as well as sources of advice, are important factors in acceptance [10]. Several reported studies indicate the level of knowledge of the parents, parental income, access to the vaccines, accurate information on vaccines and the education level of mothers played an important role in the acceptance of the vaccines [11-12]. The role of social media was also highlighted in a review exploring the characteristics of vaccine refusal of parents [13].

In this study, we explored parental factors that influence their decision making in relation to whether to have their children vaccinated or not for both non-funded and funded vaccines in Kocaeli, Turkey.

## Materials and methods

Design and Sample

The data was gathered in a semi-private pediatric polyclinic from January 2009 through March 2010 in Kocaeli, Turkey via a survey. Eligibility criteria included children aged one to four years who were coming for well-child or routine immunization visits. Parents who were present were approached in the waiting room and invited to participate in the study. Out of the 300 parents that were approached over the time scale of the study, 38 parents declined to participate in the survey. Informed consent was obtained from the parents before they filled out the survey. This study was approved by the local hospital committee.

The questionnaire was designed to explore the vaccination status of children, socio-demographic information pertinent to each participating family (such as birth date, gender, family income, parents’ education level, age, number of children and location of residential address (urban/inner-city, suburban, rural), parents’ job type, child’s immunization status and where relevant, reasons for excluding any vaccines. A total of 29 questions was included in our survey.

The survey content was based on health belief statements adopted from Taylor and Newman (2000) for each new, non-funded vaccine (pneumococcal, varicella and rotavirus vaccine) and funded vaccines. Parents indicated their level of agreement with each statement using a five-point Likert scale, with possible responses ranging from “strongly agree” to “strongly disagree.” Responses were converted to an ordinal scale with scores between one and five with one corresponding to “strongly disagree” and five corresponding to “strongly agree”.

Survey questions also investigated the opinion of parents towards new vaccines unfunded by the health insurance about whether they were willing to pay for the vaccine themselves when they acquired the knowledge about the vaccines or diseases, fear of adverse effects or insufficient information about the vaccines in the vaccination program. The parents were also asked about their reasons of acceptance of the vaccine.

Vaccination information supplied by the parents was confirmed from the child’s vaccination record book. Further checks were also carried out by consulting a central vaccination registry of the city health authority in order to remove any error in children’s vaccination status. For a child to be considered fully immunized, a child must have received all the vaccine doses in the main vaccination program and the first dose of the combined mumps and rubella (MMR).

The analysis was carried out using SPSS version 17 (IBM Inc., Chicago, Ill., USA). Frequency and percentiles were calculated and compared. Mann-Whitney test was used for comparisons, as there is no prior information regarding data distribution and the dependent variable was represented on an ordinal scale.

## Results

The questionnaires were filled out by mothers alone (67.6 %, n=177), (28.6 %, n=75) by fathers and mothers, both present, 3.1% (n=8) by fathers alone and the remaining 0.8% (n=2) were filled by other relatives. Immunization uptake among our cohort was high with 96.9% children fully immunized for nationally funded vaccines. However, only the pneumococcal vaccine was not evaluated in the full vaccination group as it was included in the nationally funded vaccination program for children born after May 2008. Three children were not immunized for MMR, five children were not immunized for BCG and one family did not know their child’s immunization status.

Among the parents who completed the survey, 8.9% indicated that the father had graduated from a college and 18.5 % university, 5.4 % indicated that the mother had graduated from a college and 13.9 % university. General social and demographic characteristics of respondents are given in Table 1.

**Table 1:** Social and demographic characteristics of respondents who reported immunization status in Kocaeli, Turkey.

Demographic factors		N=262 (%)
Income	low income	17(6.6%)
	middle income	100(38.6%)
	upper middle income	106(40.9%)
	high income	36 (13.9%)
Father's education	primary school	59 (22.8%)
	secondary school	129(49.8%)
	college	23 (8.9%)
	university	48 (18.5%)
Mother's education	primary school	110 (42.5%)
	secondary school	99 (38.2%)
	college	14 (5.4%)
	university	36 (13.9%)
Number of children	1	145 (55.6%)
	2	89(34.1%)
	>=3	27(10.4%)
Mother's job	professional/ managerial	9 (3.5%)
	skilled	28 (10.8%)
	semi-skilled	11 (4.2%)
	manual	5 (1.9%)
	other	206 (79.5%)
Father's job	professional/ managerial	46 (17.8%)
	skilled	57 (22%)
	semi-skilled	27 (10.4%)
	manual	126 (48.6%)
	other	3 (1.2%)
Father's age (in years)	18-24	5 (2.0%)
	25-34	155 (59.8%)
	35-44	95 (36.5%)
	45-54	5 (2.0%)
Mother's age (in years)	18-24	44 (16.9%)
	25-34	181 (69.6%)
	35-44	34 (13.1%)
	45-54	1 (0.4%)
Location of Residential Address	Urban/inner city	184(73%)
	Suburban	34 (13.5%)
	Rural	34(13.5%)

**Figure 1:**
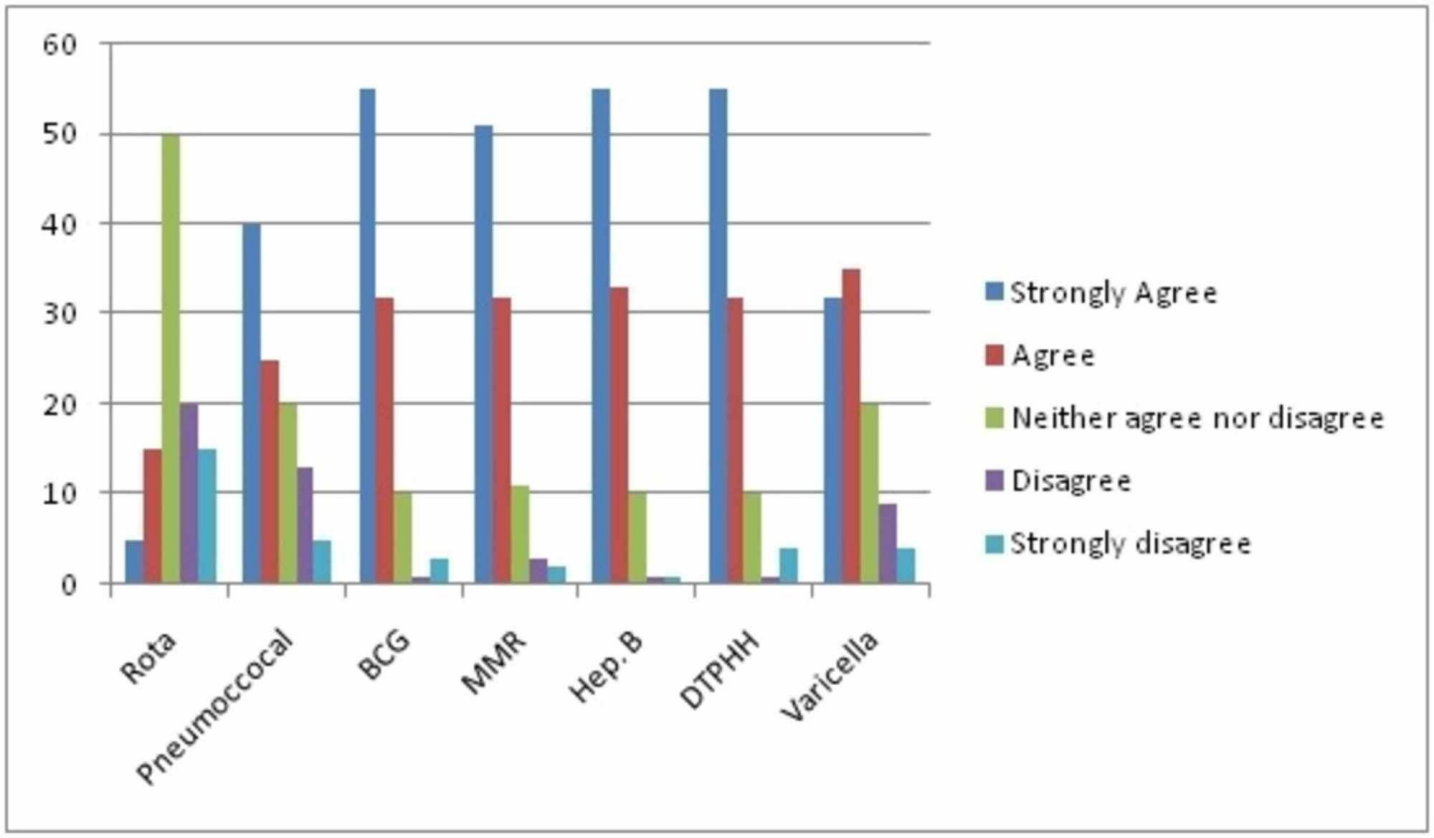
Parents’ responses to “This vaccine is unnecessary because this disease is a minor illness” for various vaccines in Kocaeli, Turkey. Scoring was as follows: strongly agree=5, agree=4 neither agree nor disagree=3, disagree=2 and strongly disagree=1

In order to assess parents’ perception of the seriousness of a disease or importance of vaccines, responses to “*This vaccine is unnecessary because this disease is a minor illness*” for each unfunded and nationally funded vaccines are shown Fig. 1. 

**Figure 2:**
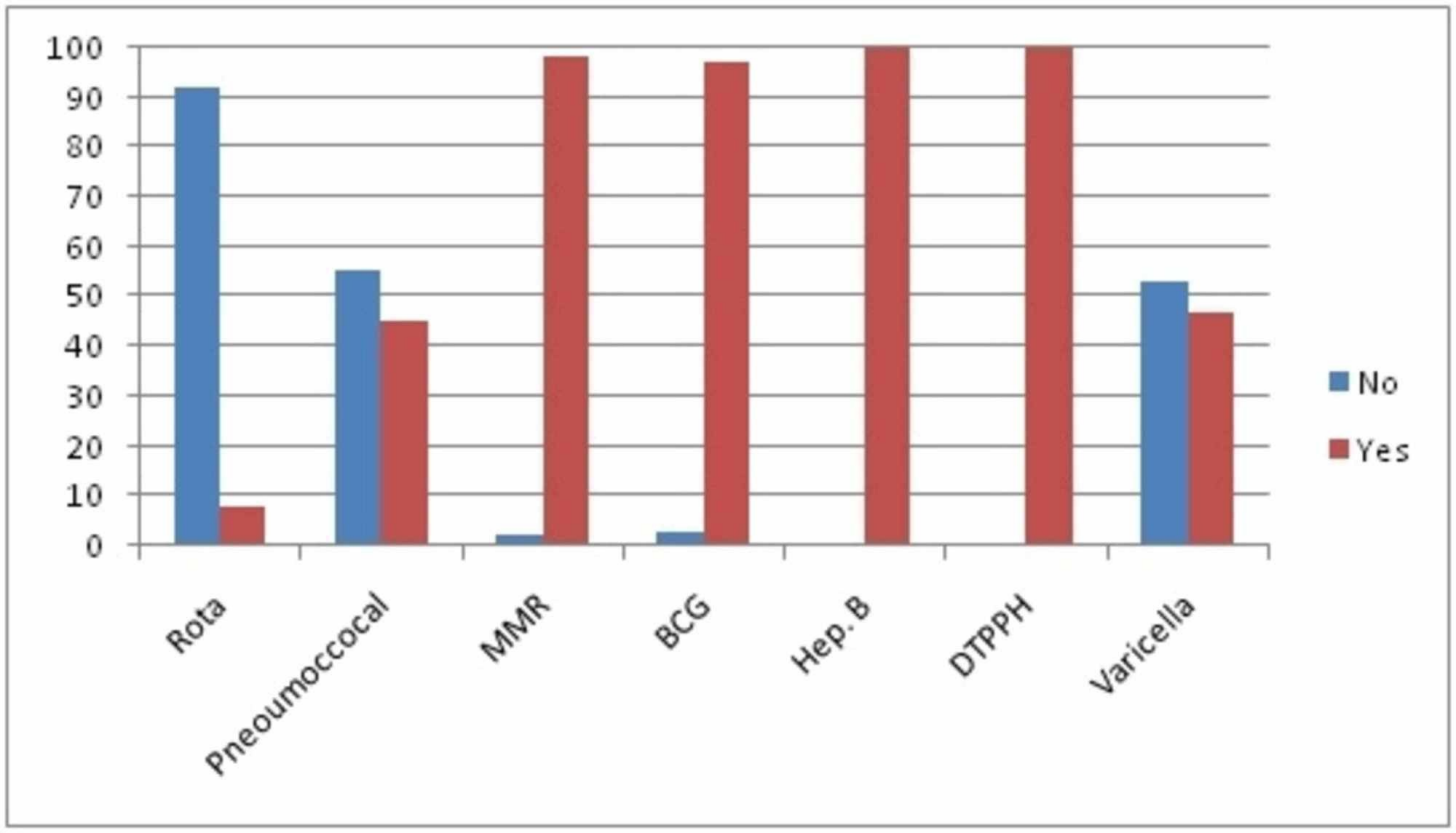
Parents’ responses to various vaccines showing the percentage of parents who would accept or decline the vaccines if they were available in the funded vaccination program in Kocaeli, Turkey.

Diphtheria, tetanus, pertussis, polio and Haemophilus Influenza type B vaccine (DTPPH.INF B) (89.3%), hepatitis B (89.7%), MMR vaccine (84.3%) and BCG (88%) were perceived to be very serious or serious by the respondents. While pneumococcal and chickenpox were viewed by the majority as less serious diseases (63.5% and 67.2%), there was no strong opinion for rotavirus virus, as parents neither agreed nor disagreed with its seriousness (50.6%) (Figure 2).

Parents’ reactions to various vaccines, showing the percentage of parents who would accept or decline the vaccines if they were available in the nationally funded vaccination program, are presented in Figure 2. DTPPH.INF B, Hep B, BCG and MMR were rated as very serious or serious by 99.6% of the parents as shown in Figure 1, and had their children vaccinated. However, 1.9% did not have BCG vaccine and 1.1% did not have the MMR vaccine for their children. While acceptance rate for pneumococcal vaccine was 56.3%, it was 53.4% for the varicella vaccine. However, only 10 children (3.8%) were immunized for rotavirus. These results are also reflected in Figure 1 as infections covered by DTPPH.INF B, Hep B, BCG, and MMR were perceived to be serious and it was important to provide protection against them.

Each parent was also asked “Are rotavirus vaccine, varicella vaccine and pneumococcal vaccine needed for your child?” Parents, who responded “yes”, mainly stated that they were positively influenced by a pediatrician for the unfunded new vaccines, as shown in Table 2.

**Table 2:** Respondents’ answers to the question “Are unfunded new vaccines (rotavirus, varicella, pneumococcal) needed for your child?” in Kocaeli, Turkey.

		N(%)	N(%)	N(%)
If no, which reasons		For varicella	For rotavirus	For pneumococcal
	Disease seriousness	0	3(1.2%)	1(0.7%)
	Cost of vaccines	76 (25.5%)	104(41.6%)	75 (51%)
	Not enough time to take children for vaccination	3 (1 % )	4 (1.6 %)	4 (2.7 %)
	No knowledge at all about vaccines	51 (17.1%)	123 (49.2%)	61 (41.5%)
	Scared of adverse effects	5 (1.7%)	12 (4.8%)	3 (2.0%)
	Insufficient information about vaccines	16 (5.4%)	4 (1.6%)	2(1.4%)
	Already has diseases	4 (1.3%)	0	1 (0.7%)
If yes, which reasons				
	Positively influenced	38 (12.8 %)	4 (40 %)	19 (16.7 %)
	Prevent diseases and complication	11 (3.7%)	2 (20%)	24 (21.1%)
	Positively influenced and to prevent diseases and its complications	94 (31.5%)	4 (40%)	71 (62.3%)

## Discussion

These findings describe a snapshot of parents’ opinions from a cohort of 262 families. Data from children with a high degree of parental income showed a similar pattern to previous reports [14-15]. Other studies have shown that in general, demographic background variables do not affect parents’ perceptions about a vaccine [16-17].

Vaccine uptake among this cohort was high and the majority expressed positive attitudes towards immunization. In general, the idea of new vaccines, particularly against life-threatening infections was perceived positively [7]. However, this study has shown that if a vaccine is promoted by the government and recommended by consultant pediatricians, the vaccine was perceived to be important and necessary.

The infections immunized by DTPPH.INF B (89.3%) and Hepatitis B (89.7%) were thought to be “very serious” or “serious” by the parents. A total of 99.6% of children were given a DTPPH.INF B and Hep B vaccine. This high uptake may be due to these vaccines being funded by the government within the vaccination program. After the decision of the Ministry of Health vaccine program to include the pneumococcal vaccine in routine immunization, its uptake rate began to increase, reaching nearly 95% in 2011.

Tetanus, diphtheria, pertussis, poliomyelitis, Hib and Hepatitis B vaccines, which are commonly administered in a hexavalent combination in the first two years of life in Germany, were thought to be very important or important by 90% of survey participants. Similarly, MMR was considered to be very important or important by 87.3% of parents [18].

Fishbein et al. (2008) reported the high cost of vaccines and the difficulty in reaching adolescents for vaccination. Our study has shown that the cost and lack of useful information had a significant impact on parental decisions for new vaccines (Table 2).

Parents did not have full knowledge about new vaccines as evident from the replies to the question ‘This vaccine is unnecessary because this disease is a minor illness’ for each new unfunded and funded vaccine (Figure 1). Figure 2 shows the percentage of the parents who would accept or decline the vaccines if they were in the nationally funded vaccination program. These results are similar to a study reported in 2007 [18]. A centrally funded public education program could help inform parents prior to launching a new vaccine in the routine immunization program. However, a recent review states that a deeper understanding of the priorities, processes, and ambivalences could be instructive to providers who work with families and those who seek to improve public health participation, particularly around vaccines [19].

One of the limitations of this study is that the study population was drawn from only one city. However, in mitigation, Kocaeli is one of the most industrialized and cosmopolitan cities in Turkey with many migrants from all corners of the country. Thus, we believe that our results will be more applicable to a larger population. Another limitation is the convenience sample together with the possibility of different responses of non-respondents.

## Conclusions

The acceptance of new vaccines depends on a complex interaction of factors, but parents’ perceptions may change dramatically if the vaccine is added to the ministry of health vaccination program by the government. The main factors influencing parents’ decisions are the lack of information and the cost. Mothers’ job types also appear to be an important factor in contrast to previous findings. The level of information, especially from a pediatrician, is also a major positive factor in the acceptance of the vaccines. It is obvious that for a new vaccination program to succeed, it should be funded by the government. In addition, our findings may reflect general parental attitudes, particularly in respect of the need for central funding and public health information availability.
